# Delayed Neurological Deficits Following Lumbar 1 Burst Fracture: A Diagnostic Challenge Without Radiological Correlates

**DOI:** 10.7759/cureus.76685

**Published:** 2024-12-31

**Authors:** Yogeshwarran Nadeson, Amir Fariz Zakaria

**Affiliations:** 1 Department of Orthopedics and Traumatology, Universiti Kebangsaan Malaysia Medical Centre, Selangor, MYS; 2 Department of Orthopedics, Spine Unit, Hospital Sungai Buloh, Sungai Buloh, MYS

**Keywords:** accurate diagnosis, clinicoradiologic mismatch, delayed neurology, medicolegal and ethics, sciwora, spinal cord injury, terminology

## Abstract

Spinal cord injuries, including rare cases without radiological abnormalities, pose diagnostic challenges, particularly in cases of delayed neurological deficit development. This case report describes a 55-year-old man with a stable L1 burst fracture who developed delayed neurological deficits two weeks after sustaining a fall despite no evidence of intrinsic or extrinsic spinal cord abnormalities on magnetic resonance imaging (MRI). The patient initially presented with back pain, normal muscle strength across all myotomes, and imaging that showed no canal stenosis or retropulsion fragments. After symptom progression to bilateral lower limb weakness, urinary incontinence, and loss of anal tone, follow-up investigations, including MRI, electromyography, nerve conduction studies, and blood tests, were unremarkable. Although the clinical presentation appeared to indicate spinal cord injury without radiological abnormality (SCIWORA), the delayed onset of symptoms and structural abnormalities in this case fell outside traditional SCIWORA criteria. This case highlights a gap in the current terminology used to describe spinal cord injuries with delayed neurological presentation, emphasizing the need for more precise classification to inform diagnosis, management, and medicolegal documentation.

## Introduction

Spinal cord injuries are common in orthopedic and neurosurgical trauma, and imaging modalities, particularly magnetic resonance imaging (MRI), are vital in correlating clinical findings and guiding treatment. Spinal cord injury without any obvious radiological abnormality (SCIWORA) [1] is a rare diagnosis, often made by exclusion, and is more common in children. Pang and Wilberger first introduced the term SCIWORA in 1982 [2] to describe this condition in pediatric patients. Over time, the term was adapted for adults due to a lack of appropriate terminology for similar cases in adult populations.

The term “adult SCIWORA” [3] is often misunderstood and misapplied in clinical practice to describe a variety of spinal cord injuries. Several variations have emerged to address specific clinical findings, including spinal cord injury without radiologic evidence of trauma (SCIWORET) and spinal cord injury without computed tomography (CT) evidence of trauma (SCIWOCTET) [4]. In cases where MRI findings are negative, the term spinal cord injury without neuroimaging abnormality (SCIWONA) is used. However, terminology remains inadequate for situations where there is CT or MRI evidence of structural trauma but no intrinsic or extrinsic cord abnormalities, and the patient develops delayed neurological deficits. This report highlights such a case, describing a patient with delayed neurological symptoms despite the absence of significant spinal cord abnormalities on MRI.

## Case presentation

**Figure 1:**
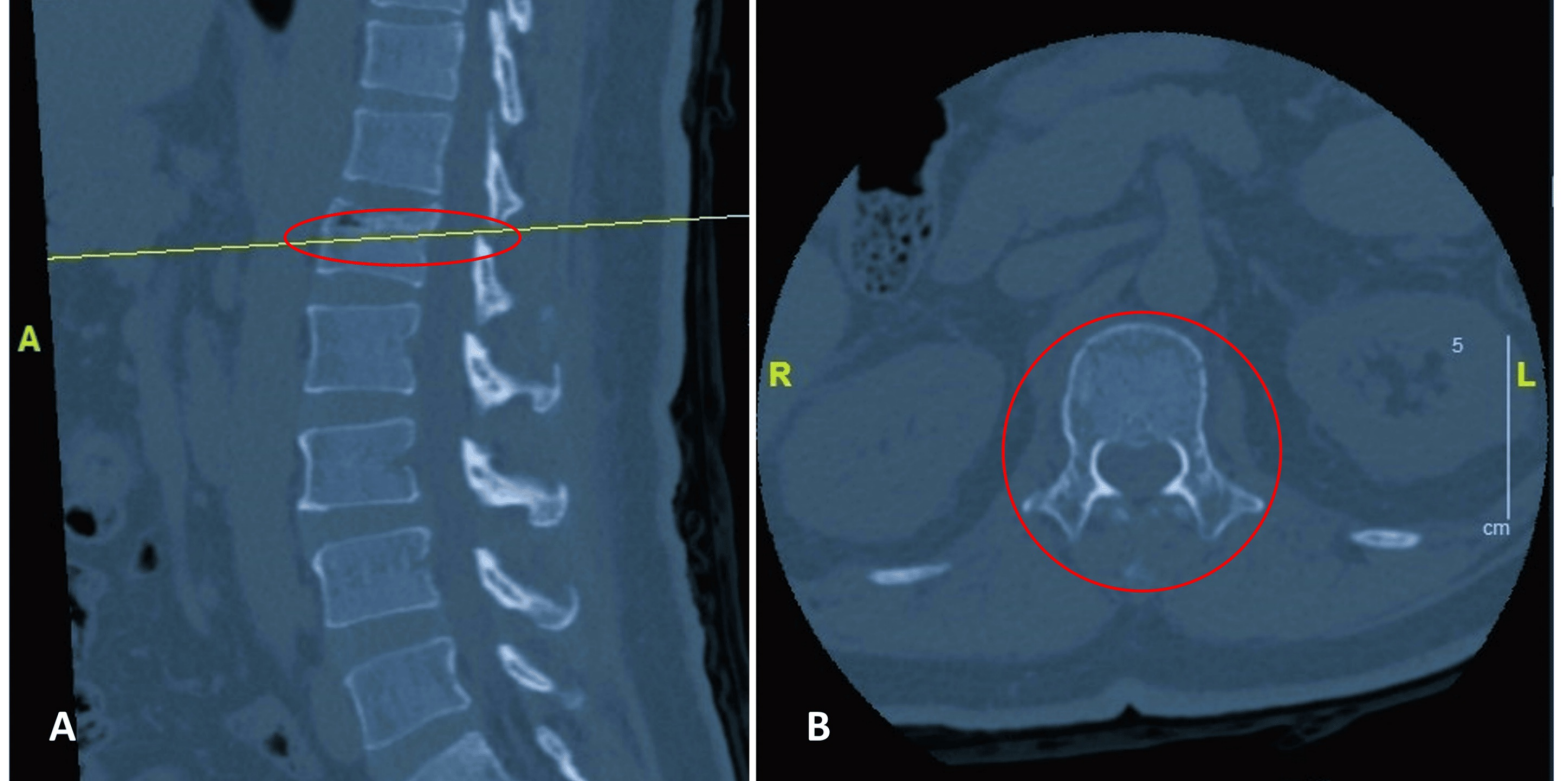
A: Sagittal view indicates the spinal cord level where the CT slice was taken. B: CT scan of the lumbar spine shows an L1 burst fracture classified as A3 according to the comprehensive classification system for spinal injuries CT: computed tomography

A 55-year-old man with a history of hypertension presented to our center following a fall in September 2023 during a workplace event. After the trauma, the patient experienced back pain but was still able to walk. Upon initial examination, muscle strength was graded 5 across all myotomes, and tenderness was noted in the lower lumbar region. A CT scan of the lumbar spine (Figure 1) revealed an L1 burst fracture, classified as type A3 according to the Arbeitsgemeinschaft für Osteosynthesefragen's (AO) comprehensive classification system for spinal injuries without canal stenosis and retropulsion fragments.

After initial pain management, the patient was discharged with a body cast and prescribed analgesics. Two weeks later, he developed progressive bilateral lower limb weakness accompanied by urinary incontinence. One month post-trauma, neurological examination revealed muscle strength graded 0 across all lower limb myotomes, with diminished sensation from the L2 level onward. Reflexes were decreased in both lower limbs; however, they were normal over the bilateral upper limb. A rectal examination revealed a lax anal tone and the absence of voluntary anal contraction.

**Figure 2:**
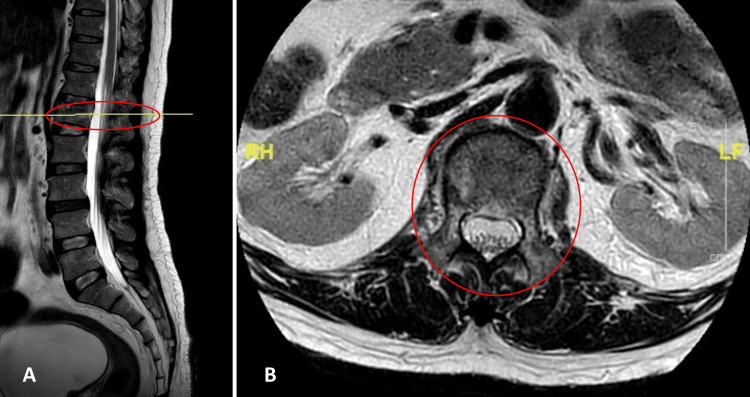
A: Sagittal view indicates the spinal cord level where the MRI was taken. B: T2-weighted MRI of the thoracolumbar spine shows no significant cord edema or changes in fracture morphology MRI: magnetic resonance imaging

**Figure 3:**
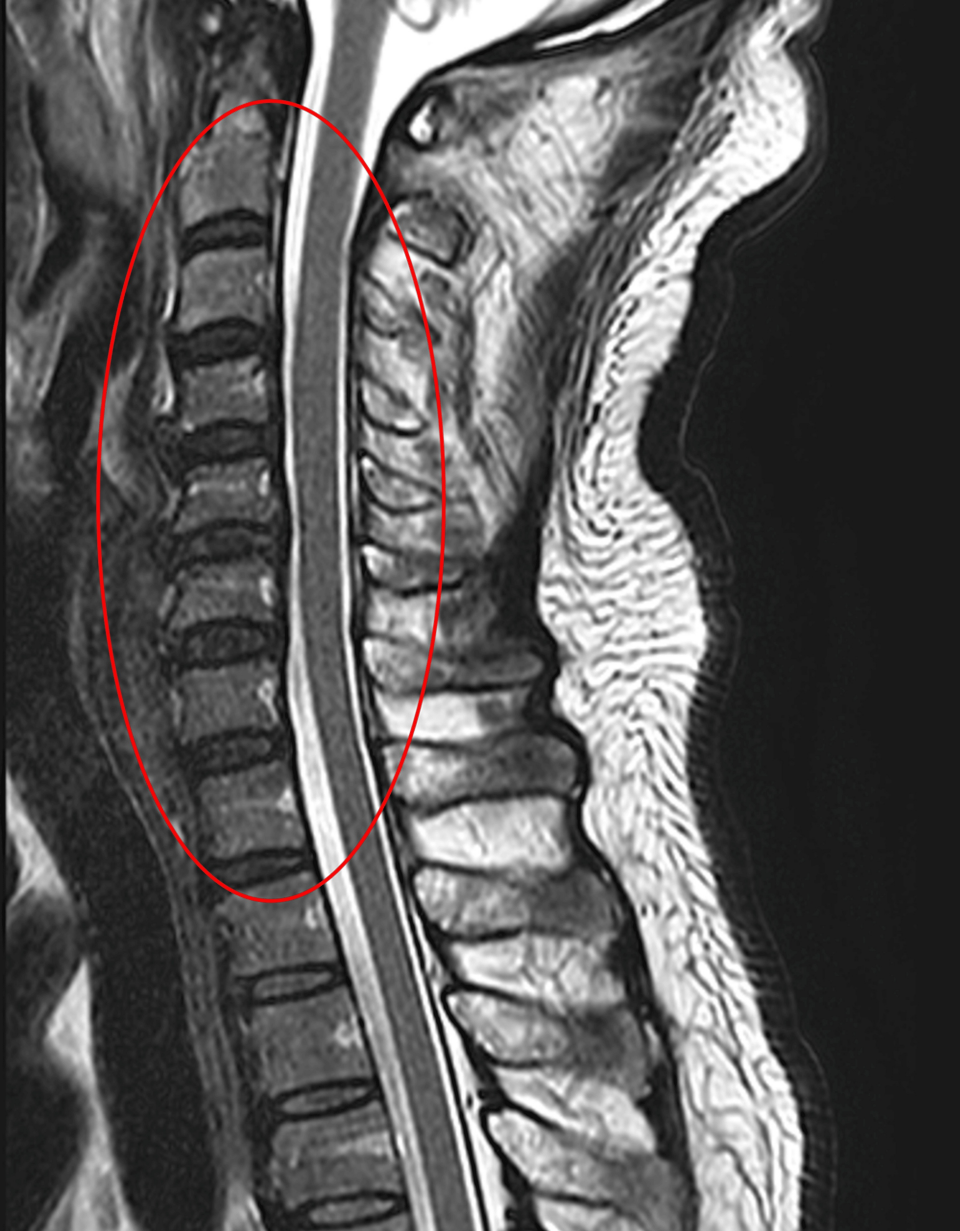
Sagittal T2-weighted MRI of the cervical region (circled) demonstrating the absence of intrinsic or extrinsic spinal cord abnormalities MRI: magnetic resonance imaging

Follow-up MRI of the entire spine (Figures 2-3) revealed no cord edema or fracture morphology changes. Electromyography and nerve conduction studies revealed no abnormalities, and repeated blood tests, including tumor markers, were unremarkable. A contrast-enhanced CT scan of the brain also showed no abnormalities. On follow-up in the outpatient clinic, the patient showed gradual recovery, with muscle strength graded 2 at the L2 and L3 myotomes but remaining 0 from L4 downward.

## Discussion

This patient presented with a stable burst fracture without significant canal stenosis or retropulsion fragments [5]. The primary challenge arose from the delayed onset of neurological symptoms one month after the initial trauma, without radiological or electrophysiological evidence of spinal cord or cortical injury. The mismatch between clinical progression and imaging findings further complicated the diagnosis.

Delayed neurological deficits are rare in thoracolumbar fractures [6], presumably attributable to ample spinal canal dimensions, and while the clinical presentation initially suggested SCIWORA, the criteria for diagnosing SCIWORA were not fulfilled [2,3]. Classical cases of SCIWORA involve neurological symptoms within 48 to 72 hours after injury, with no structural abnormalities observed on X-ray-based imaging.

A specific study [7] documented an instance of SCIWORA involving concomitant thoracic and lumbar injuries in 2012, wherein an adult exhibited delayed thoracic myelopathy onset on the seventh-day post-trauma, with initial MRI findings revealing wedge-shaped vertebrae at the thoracic 12 and L1 levels, yet no indications of spinal cord injury, akin to the presentation of our patient. Subsequent MRI conducted on day 17 similarly disclosed no abnormal thoracic lesions upon formal evaluation, although there was a suspicious focal signal alteration that could suggest intramedullary edema, which was absent in our patient even upon repeated MRI assessment.

Another publication in 2022 [8] reported a case of delayed SCIWORA with clinical manifestations of paraplegia analogous to those exhibited by our patient. Nevertheless, in contrast to our patient, symptoms in this case emerged within 72 hours following the injury, with initial MRI assessments indicating spinal cord abnormalities from thoracic 9 to thoracic 11, while vertebral injury was not observed. In comparison, our patient did not present any spinal cord abnormalities but exhibited non-compressive lumbar vertebral injury and experienced neurological symptoms at a considerably later stage post-initial trauma.

The two cases underscore the complexities encountered in categorizing patients with SCIWORA diagnoses in clinical practice. There remains an absence of consensus regarding the temporal criteria for SCIWORA diagnosis in adults, as the existing literature indicates a variable timeline for neurological symptom development extending up to seven days, whereas our patient experienced a delay of two weeks. Pang, who revisited his seminal 1982 publication in 2004 [9], proposed that only cases exhibiting neural injuries with normal MRI findings should be classified as SCIWORA, explicitly excluding purely extraneural compressive lesions from this definition. It is important to acknowledge, however, that this article predominantly pertains to the pediatric demographic. Novice trainees or even experienced surgeons may inadvertently apply incorrect terminology in such cases.

This case highlights the limitations of existing terminology, which fails to account for delayed neurological symptoms in patients with structural abnormalities but no visible spinal cord injury. The absence of an appropriate classification for such cases indicates the need for more precise terminology to avoid miscommunication and ensure accurate diagnosis. Clear documentation is critical, given the rise in medicolegal cases and associated costs [10]. In this context, the above presentation could be easily misdiagnosed as SCIWORA by residents or even junior surgeons. Therefore, when diagnosing patients, clinicians should remain vigilant when diagnosing atypical presentations to reduce miscommunication and legal risk.

## Conclusions

This case illustrates a gap in the current terminology used to classify spinal cord injuries. In an era marked by increasing litigation and medicolegal scrutiny, accurate documentation of diagnoses is essential. We recommend clinicians exercise caution when assigning diagnostic labels, especially in atypical cases, to reduce the risk of misdiagnosis and ensure appropriate care.

## References

[1] Atesok K, Tanaka N, O'Brien A (2018). Posttraumatic spinal cord injury without radiographic abnormality. Adv Orthop.

[2] Pang D, Wilberger JE Jr (1982). Spinal cord injury without radiographic abnormalities in children. J Neurosurg.

[3] Dreizin D, Kim W, Kim JS, Boscak AR, Bodanapally UK, Munera F, Stein DM (2015). Will the real SCIWORA please stand up? Exploring clinicoradiologic mismatch in closed spinal cord injuries. AJR Am J Roentgenol.

[4] Como JJ, Samia H, Nemunaitis GA, Jain V, Anderson JS, Malangoni MA, Claridge JA (2012). The misapplication of the term spinal cord injury without radiographic abnormality (SCIWORA) in adults. J Trauma Acute Care Surg.

[5] Woo JH, Lee HW, Choi HJ, Kwon YM (2021). Are "unstable" burst fractures really unstable?. J Korean Neurosurg Soc.

[6] Kanna RM, Khurjekar K (2018). Thoracolumbar trauma with delayed presentation. Indian Spine J.

[7] Park MC, Bok SK, Lee SJ, Ahn DH, Lee YJ (2012). Delayed onset of thoracic SCIWORA in adults. Ann Rehabil Med.

[8] Dubey A, Tomar S, Gupta A, Khandelwal D (2018). Delayed paraplegia in an adult patient with spinal cord injury without radiographic abnormality of dorsal spine: a lesson learned. Asian J Neurosurg.

[9] Pang D (2004). Spinal cord injury without radiographic abnormality in children, 2 decades later. Neurosurgery.

[10] Gathen M, Jaenisch M, Fuchs F (2022). Litigations in orthopedics and trauma surgery: reasons, dynamics, and profiles. Arch Orthop Trauma Surg.

